# Polygenic disruption of retinoid signalling in schizophrenia and a severe cognitive deficit subtype

**DOI:** 10.1038/s41380-018-0305-0

**Published:** 2018-12-07

**Authors:** William R. Reay, Joshua R. Atkins, Yann Quidé, Vaughan J. Carr, Melissa J. Green, Murray J. Cairns

**Affiliations:** 1 School of Biomedical Sciences and Pharmacy, The University of Newcastle, Callaghan, NSW Australia; 2 Centre for Brain and Mental Health Research, Hunter Medical Research Institute, Newcastle, NSW Australia; 3 School of Psychiatry, University of New South Wales, Sydney, NSW Australia; 4 Neuroscience Research Australia, Sydney, NSW Australia; 5 Department of Psychiatry, Monash University, Melbourne, VIC Australia

**Keywords:** Schizophrenia, Genetics

## Abstract

Retinoid metabolites of vitamin A are intrinsically linked to neural development, connectivity and plasticity, and have been implicated in the pathophysiology of schizophrenia. We hypothesised that a greater burden of common and rare genomic variation in genes involved with retinoid biogenesis and signalling could be associated with schizophrenia and its cognitive symptoms. Common variants associated with schizophrenia in the largest genome-wide association study were aggregated in retinoid genes and used to formulate a polygenic risk score (PRS_Ret_) for each participant in the Australian Schizophrenia Research Bank. In support of our hypothesis, we found PRS_Ret_ to be significantly associated with the disorder. Cases with severe cognitive deficits, while not further differentiated by PRS_Ret_, were enriched with rare variation in the retinoic acid receptor beta gene *RARB*, detected through whole-genome sequencing. *RARB* rare variant burden was also associated with reduced cerebellar volume in the cases with marked cognitive deficit, and with covariation in grey matter throughout the brain. An excess of rare variation was further observed in schizophrenia in retinoic acid response elements proximal to target genes, which we show are differentially expressed in the disorder in two RNA sequencing datasets. Our results suggest that genomic variation may disrupt retinoid signalling in schizophrenia, with particular significance for cases with severe cognitive impairment.

## Introduction

Schizophrenia is a complex disorder that likely emerges from an array of genetic and environmental influences [1, 2]. Large-scale case–control genotyping initiatives have revealed that both common and rare variants are associated with the disorder, across the entire genome [1, 3, 4]. Although schizophrenia is highly heritable, genetic heterogeneity is likely to contribute to significant variation in clinical presentation and outcome. The genomic architecture unique to distinct clinical sub-phenotypes may therefore not be revealed in case–control study designs, thereby obscuring the detection of novel treatment targets for patients with different phenotypic profiles. This is particularly evident in the cognitive dimension of the disorder, for which genetic liability is yet to be characterised and the cognitive symptoms remain relatively resistant to treatment. Cognitive impairment in schizophrenia typically manifests before psychosis and is highly variable among cases, with evidence of a subtype of schizophrenia patients with severe cognitive deficits (CDs) [5, 6]. Distinct cognitive phenotypes in schizophrenia show some evidence of differential grey matter abnormalities, and loss of dendritic arborisation and connectivity, possibly reflecting its neurodevelopmental origins [7, 8], and may therefore be associated with distinct genetic architecture that is yet to be fully elucidated [5, 6, 9].

Many aspects of neurodevelopment, such as neural differentiation, are regulated by retinoids, and previous studies have implicated these molecules, or their signalling apparatus, as relevant to the pathogenesis of schizophrenia [10, 11]. Retinoids are metabolites of vitamin A (all-*trans* retinol [at-ROL]) – hereafter referred to as retinol, a fat-soluble vitamin derived from food in active form or as pro-vitamin precursors such as beta-carotene. The most biologically active retinol metabolite, all-*trans* retinoic acid (at-RA), exerts control over the expression of thousands of transcripts genome wide [12, 13]. In the canonical mechanism, at-RA associates with nuclear receptors bound to sequence motifs termed retinoic acid response elements (RAREs), one of the most well-characterised configurations being two repeats of a hexameric motif separated by five nucleotides (DR5-RARE) [12]. The active nuclear receptor complex induces recruitment of co-factors that influence the expression of associated genes. Retinoid signalling is intrinsically linked to neurogenesis in utero along with neuronal homeostasis in the mature brain [14, 15]. Although there is compelling support for the dysregulation of retinoid signalling during neurodevelopment in schizophrenia, including reports of low maternal retinol during gestation [16], several genes in this pathway remain differentially expressed in post-mortem samples [17, 18]. This pathway has also been implicated in synaptic plasticity [15], suggesting the impact on brain function and behaviour persists in adults. Promising initial clinical trials of a retinoid X receptor agonist, Bexarotene, further supports that modulation of retinoid signalling can have a clinically significant impact on schizophrenia symptoms [19, 20]. Given the heterogeneity of the disorder, it is plausible that retinol deficiency or genomic dysregulation of retinoid signalling could influence the response to this treatment and other retinoid modulating compounds.

Progress in identifying variants that may disrupt at-RA functionality in schizophrenia has recently been facilitated by collaborative genome-wide association studies (GWAS) assembled by the Psychiatric Genomics Consortium (PGC). In the largest PGC mega analysis of schizophrenia, over 100 loci were uncovered at rigorous genome-wide significance levels [1]. Interestingly, five genes involved in retinoid biology are located in genome-wide significant loci (Table 1). The polygenic impact of many single-nucleotide polymorphisms (SNPs) not exceeding stringent genome-wide statistical correction are also regarded as clinically significant [21], and likely important for understanding the significance of at-RA genes in the disorder. Exome sequencing has also revealed an enrichment of rare variation in schizophrenia, which is predicted to have a higher effect size than common SNPs due to purifying selection [3, 4, 22]. However, to date, genomic analyses have largely been limited to traditional nosological categories and the role of rare non-coding variation is yet to be considered. Non-coding regions proposed to influence neural complexity are a rich source of RAREs and likely play an integral role in at-RA signalling [23]. Whole-genome sequencing (WGS), with its capacity to capture rare variants genome wide, offers promise to uncover functionally significant loci in schizophrenia associated with clinical subtypes. WGS is particularly advantageous as it facilitates the detection of non-coding variation in regions missed by exome sequencing, which may disturb regulation of gene expression.

**Table 1:** Marker SNPs from the 2014 psychiatric genomics consortium schizophrenia mega-GWAS within a haplotype to which a retinoid-related gene was mapped

SNP ID	Annotation	Retinoid gene
rs34269918	Intron variant	*RERE*
rs2053079	Intron variant	*ZNF536*
rs3768644	Intron variant	*CYP26B1*
rs8082590	Intron variant	*RAI1*^a^
rs12325245	Intergenic variant	*CNOT1*

^a^Marker SNP (rs8082590) located within *GID4* gene but implicated haplotype features the retinoic acid induced 1 (*RAI1*) locus

Given that disruption of retinoid biology should alter neurodevelopment, we hypothesised that retinoid signalling dysregulation plays a role in the emergence of more severe CDs and adverse clinical outcomes in schizophrenia. In this study, we therefore sought to integrate the polygenic effect of common and rare variation, using systems biology to investigate at-RA signalling, in a cohort of schizophrenia cases that have previously been stratified by multiple cognitive measures to highlight a CD subtype [5]. We observed that common polygenic risk in retinoid genes implicated by GWAS was associated with schizophrenia, but not overrepresented in the CD group. However, rare variation in retinoid genes was only enriched in schizophrenia cases with severe CD, with the retinoic acid receptor beta gene *RARB* significantly associated with this subtype. Increasing burden of rare *RARB* variation was also associated with decreased grey matter volume in a left posterior cerebellar region in the CD subgroup after voxel-wise correction, along with covariation amongst grey matter concentration (GMC) in several brain regions. Analysis of non-coding sequence in DR5-RARE, proximal to genes, revealed an excess of rare variation that could disrupt the expression of retinoid target transcripts. This, and other forms of retinoid dysregulation, was supported by differential expression of DR5-RARE proximal genes in two independent schizophrenia cohorts, which were enriched in functionally significant pathways.

## Materials and methods

### Participants

Participants in this study were sourced from the Australian Schizophrenia Research Bank (ASRB), which is a public bank of clinical and cognitive data, including structural magnetic resonance imaging (MRI) scans and DNA samples, for a large cohort of schizophrenia cases and controls, collected across five cooperating sites in Australia [24]. Detailed descriptions of the exclusion criteria and procedures for consent have been outlined elsewhere [5, 24]. The use of these data was approved by the University of Newcastle Human Ethics Research Committee (HREC) and the Australian Schizophrenia Research Bank. First, ASRB schizophrenia cases and non-neuropsychiatric controls with no immediate family history of psychoses were selected for SNP genotyping. Individuals were excluded during quality control (QC) as described in supplementary note 1, with 425 schizophrenia cases and 251 controls retained for analysis. The case cohort was comprised of 283 males (67%), whereas controls were 43% male (Supplementary Table 5, 6). WGS was performed on a subset of the ASRB for whom clinical and cognitive data were previously collected, with 469 samples available for this study, consisting of 321 affected individuals and 148 controls (Supplementary Table 7). In the WGS cohort, MRI data were also accessed for schizophrenia cases as available (*N* = 210, Supplementary Table 8). Data are available upon application to the ASRB (https://www.neura.edu.au/discovery-portal/asrb/).

As described in Green et al. [5], the multidimensional Grade of Membership (GoM) clustering technique was used to derive subgroups of cognitive performance in the ASRB schizophrenia cohort with available cognitive data. Briefly, nine cognitive measures were utilised as input into the GoM (Supplementary Table 9). The most parsimonious GoM model partitioned schizophrenia cases into the CD subtype, with a greater degree of global cognitive impairment, and a cognitively spared (CS) subtype whose performance was intermediate to the CD subtype and healthy controls. The distribution of the cognitive subtypes in the array-genotyped, WGS and MRI, cohorts are presented in Supplementary Table 6, 7 and 8, respectively.

### Genotyping and sequencing

Investigators were blinded to phenotype during processing of samples for genotyping and/or sequencing. Genomic DNA from peripheral blood mononucleocytes was extracted for SNP genotyping and/or WGS. SNP genotyping utilised the Illumina Infinitium Human 610K (610-Quad) BeadChip in accordance with standard manufacturer protocols. Pre-imputation QC on autosomal array SNPs and subject exclusion criteria is outlined in supplementary note 1. After phasing haplotypes with 10 megabase chunks and 500 kilobase (kb) overlap with Eagle 2.3.2 [25], imputation was performed on loci which passed QC using Minimac3 default parameters and the 1000 Genomes Phase 3 European reference panel [26]. High-quality SNPs post-imputation were retained for analysis (INFO score > 0.8, missingness < 2%, *N*_SNPs_ = 7,199,582).

The ASRB subset, which underwent WGS, was sequenced on the Illumina HiSeq X Ten platform (Supplementary Note 2). Bowtie2 aligned raw reads to the hg19 reference genome, followed by conversion of SAM files to binary BAM files via SAMtools [27]. Duplicate reads were removed using Picard tools (https://broadinstitute.github.io/picard/). Quality score recalibration and indel realignment were undertaken with the GATK v3.4 tools *BaseRecalibrator* and *IndelRealigner*, respectively [28]. Retinoid loci selected for analysis in this study were called using the GATK *HaplotypeCaller* framework, followed by *GenotypeGVCFs*. Variant filtering was applied, adapted from GATK best practices (Supplementary Table 11), and after importing data into the genome analysis tool KGGseq, an additional battery of QC was applied by that tool on retained variants as described elsewhere [29]. Only rare sequenced sites were analysed in this study, with a frequency threshold implemented of <0.01% (minor allele frequency [MAF] < 1 × 10^−4^) in the genome aggregation database (gnomAD).

### Selection of retinoid loci

To investigate the impact of genomic variation on at-RA signalling in schizophrenia, we constructed a gene panel of 107 retinoid genes (Supplementary Note 3). Variants, which may disrupt retinoic acid receptor binding, were analysed by obtaining the coordinates of in silico predicted DR5-RARE within 10 kb of a gene from Lalevée et al. [12], updated to hg19 assembly via LiftOver (https://genome.ucsc.edu/cgi-bin/hgLiftOver).

### Aggregation of schizophrenia GWAS variants in retinoid genes

**Fig. 1:**
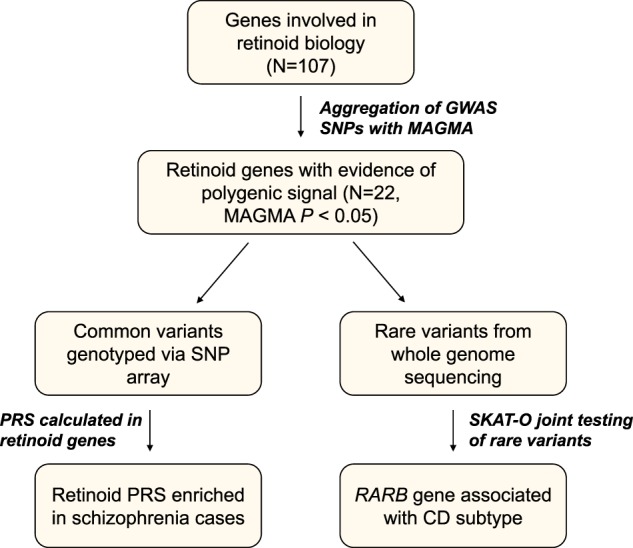
Overview of investigation of the impact of common and rare variation in retinoid genes. Using gene ontology and the wider literature, a panel of 107 genes involved with retinoid biology were collated and tested for the aggregated effect of GWAS SNPs using MAGMA. An uncorrected threshold of *P* < 0.05 was used to delineate genes with evidence of a polygenic effect. Polygenic risk score (PRS) was then calculated in individuals for this retinoid panel. Retinoid PRS was associated with schizophrenia but did not disproportionately affect the cognitive deficit (CD) subtype. Rare variant association (MAF < 0.01%) from whole-genome sequencing was tested at gene level for each of the 22 genes using the SKAT-O framework. The retinoic acid receptor gene *RARB* was significantly associated with the CD population, but there were no significant gene level signals for the full case cohort (cognitively mixed) relative to controls

SNPs from the 2014 PGC schizophrenia GWAS were analysed for enrichment in each retinoid-related gene from the panel using Multi-Marker Analysis of Genomic Annotation (MAGMA) [30]. Briefly, *P*-values for SNPs mapped to retinoid genes are aggregated and their combined significance tested to derive a gene level measure of significance. Linkage disequilibrium between markers was accounted for in this framework with the 1000 Genomes Phase 3 European reference panel. Genes that demonstrated nominally significant association (*P*_Uncorrected_ < 0.05), were selected to test association of common and rare variant enrichment in the ASRB cohorts as they display evidence of a potential polygenic effect (Fig. 1). Tissue-specific expression of genes below the polygenic threshold relative to the rest of the retinoid panel was investigated using the GENE2FUNC component of FUMA [31]. A two-sided Fisher’s exact test tested the overrepresentation of these genes as loss-of-function intolerant (pLI > 0.9) [32]. The same approach was used to investigate enrichment of de novo variation in neuropsychiatric disorders (Possible, *P* *<* 0.05) as characterised in the neuropsychiatric de novo mutation database (NPdenovo, Supplementary Table 10) [33]. NPdenovo curated established de novo variants from sequencing studies across four neuropsychiatric phenotypes (schizophrenia, autism spectrum disorders, intellectual disability and epileptic encephalopathy), and calculated gene-wise association of de novo variation with each of the disorders (http://www.wzgenomics.cn/NPdenovo/).

### Polygenic risk score in retinoid genes implicated by GWAS

Genome-wide polygenic risk score (PRS) was calculated for each subject in the array cohort using the PGC2 schizophrenia GWAS summary statistics with PRSice-2 default parameters [34]. The flag *--fastscore* calculated PRS at eight *P*-value thresholds (*P*_T_) – *P*_T_ = 1 × 10^−5^, 1 × 10^−3^, 0.05, 0.1, 0.2, 0.3, 0.5. Enrichment of PRS in schizophrenia was tested relative to controls, as well as between the two schizophrenia cognitive subtypes (CD/CS), using binomial logistic regression adjusted for sex and the first three principal components. Nagelkerke’s *R*^2^ was utilised to select the *P*_T_, which fit best in each model. Variants mapped to the 22 retinoid genes with evidence of gene level association (MAGMA *P* < 0.05) were extracted to construct a retinoid PRS (PRS_Ret_). The association of PRS_Ret_ in schizophrenia relative to controls and, in CD cases relative to CS, was tested using the same logistic regression model but additionally covaried for genome-wide PRS, which was depleted of variants mapped to the 22 retinoid genes. We assessed enrichment of PRS_Ret_ using a *χ*^2^ test of residual deviance between two logistic regression models constructed with the glm function in R version 3.3.3. The models are outlined below where *y* is affection status, i.e., either case vs control or CD vs CS.

1 $$y\sim \mathrm{Sex} + \mathrm{PCs} + \mathrm{Total}\,\mathrm{PRS}$$

2 $$y\sim \mathrm{Sex} + \mathrm{PCs} + \mathrm{Total}\,\mathrm{PRS} + \mathrm{PRS}_{\mathrm{Ret}}$$

The variance explained by both total PRS (depleted for PRS_Ret_) and PRS_Ret_ was reported on the liability scale assuming a 1% population prevalence of schizophrenia [35]. In the case of PRS_Ret_, *R*^2^ was computed in model 2 using model  1 as the null.

### Rare variant association

Association between rare variants in the 22 retinoid PRS risk genes with schizophrenia, and also the CD subtype separately, was determined using the SKAT-O (Optimal SNP-Set (Sequence) Kernel Association Test) methodology. The SKAT-O model was constructed in the SKAT R package with sex as a covariate and small sample size adjustment as described previously [36]. Bonferroni correction was applied for the number of independent tests (22 genes tested in two phenotypes) using the *p.adjust* function in R. Temporal expression data during development for *RARB* was sourced from BrainSpan, a database with expression of genes at discrete timepoints throughout the development and lifespan of the human brain, and visualised to anatomical regions with the CerebroViz package [37, 38].

### MRI

MRI images were collected by the ASRB from five Australian research sites and images processed using standard protocols (see Supplementary Note 4). The distribution of usable scans (*N* = 210) for schizophrenia patients from the WGS cohort were statistically uniform across scanning site locations when comparing CD and CS (*χ*^2^ = 3.959, *df* = 4, *P* = 0.412). Total intracranial volume (TIV), white matter volume (WMV), grey matter volume (GMV) and cerebrospinal fluid (CSF) volume was extracted using the Computational Anatomy Toolbox (CAT12, v1073; Structural Brain Mapping Group, Jena University Hospital, Jena, Germany; http://dbm.neuro.uni-jena.de/cat/index.html) for SPM12 (v6906; Wellcome Trust Centre for Neuroimaging, London, UK; http://www.fil.ion.ucl.ac.uk/spm) in MATLAB r2013a (Mathworks Inc., Sherborn, MA, USA).

Voxel-based morphometry (VBM) was performed to assess the impact of rare variant burden in the significant *RARB* gene. After pre-processing (Supplementary Note 4), the effect of cognitive group (CD/CS) on total GM, WM and CSF measures was investigated using multivariate analysis of covariance (MANCOVA). All models in the imaging analyses were adjusted for sex, age, TIV and scanning site. The individual burden of rare *RARB* burden was derived using the allelic scoring flag (*--score*) in PLINK 1.9 [39]. A hierarchical multiple regression was conducted on whole brain volumes with cognitive group, *RARB* rare variant burden and their interaction as independent variables. Global scaling was used with the TIV option to reduce the effect of orthogonality due to strong correlation between TIV and the other model covariates, implicating a change in the absolute threshold for masking (0.2/30 = 0.007). A stringent voxel-wise threshold of *P*(FWE_Corr_) < 0.05 (family-wise error [FWE] corrected) was used for statistical inference, and only clusters larger than 30 voxels (around 1 cm^3^) were considered.

Source-based morphometry (SBM) investigated the effect of *RARB* rare variants on variation amongst brain regions with correlated GMC, termed ‘source’ regions (Supplementary Note 4). The SBM protocol is outlined in detail in Xu et al. [40]. Briefly, independent components analysis (ICA) was conducted on pre-processed grey matter images using the Infomax algorithm as part of the “Group ICA for fMRI Toolbox” (GIFT v4.0a; http://mialab.mrn.org/software/gift) to derive these regions of correlated GMC, with 36 components estimated by ICA. We sought to identify if any GMC covariation in ICA components were associated with *RARB* rare variant burden in each cognitive subtype using a series of hierarchical regressions; wherein *RARB* burden was the dependent variable, the VBM covariates entered in the first step, followed by the 36 components. The interpretation of the effect of *RARB* rare variation on significantly associated components is dependent on the voxels, which comprise that region and the direction of effect of the regression coefficient (Supplementary Table 13, 14).

### DR5-RARE analyses

Coordinates for automsomal in silico predicted DR5-RARE within 10 kb of a transcription start site were sourced from Lalevée et al. [12]. Enrichment of rare variation in DR5-RARE was quantified, and enrichment tested in schizophrenia cases using a two-sided Fisher’s exact test, whereby variants were randomised amongst the two cohorts so that no one sample had greater than two variants. Common SNPs from the PGC2 schizophrenia GWAS, which overlap a predicted motif, were extracted. Further, genes from within 10 kb of a predicted DR5-RARE were sourced from the summary statistics of two published schizophrenia RNAseq studies in the brain and blood (Supplementary Note 5) [17, 41].

## Results

### Schizophrenia GWAS variants are enriched in retinoid genes

**Fig. 2:**
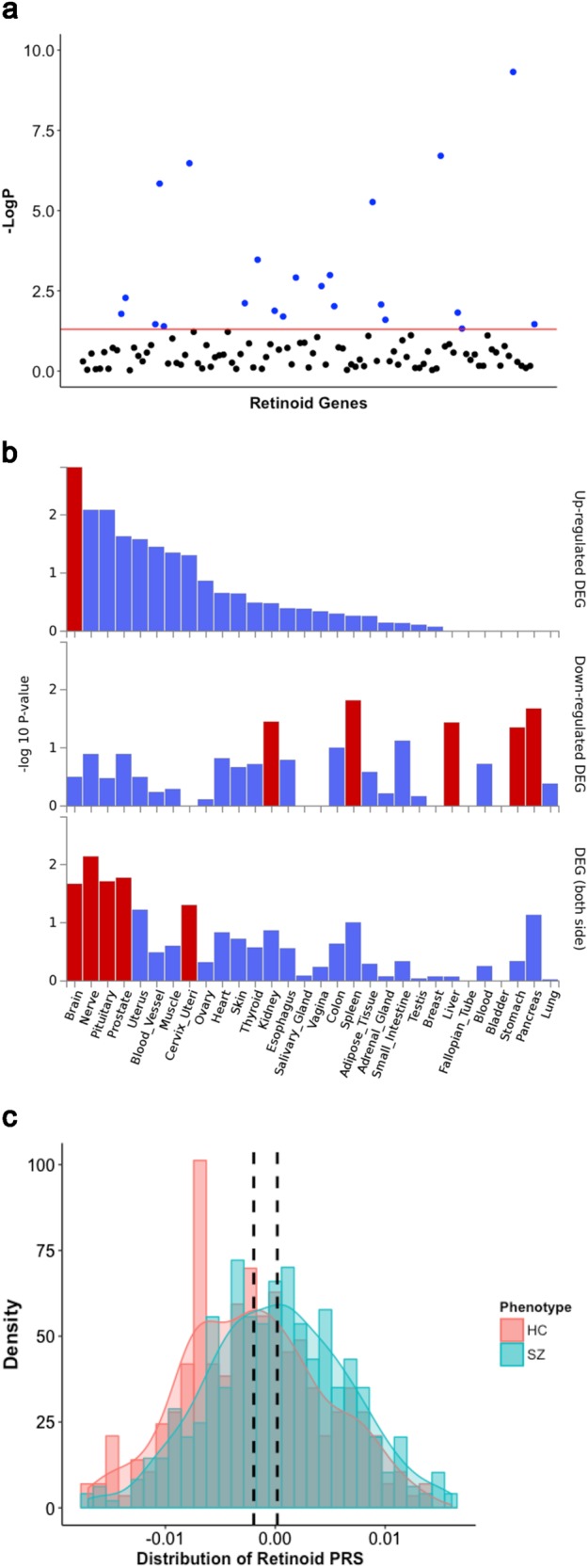
GWAS variant enrichment in retinoid genes and their relative tissue specificity. **a** Enrichment of schizophrenia GWAS variants in retinoid genes, genes highlighted blue are below the threshold of nominal uncorrected significance (*P* < 0.05). **b** Tissue-specific expression of 22 significant MAGMA genes relative to the rest of the retinoid panel. Bars highlighted red are statistically significant after multiple testing correction. One-sided (for upregulation and downregulation) and two-sided tests are reported. DEG = differentially expressed genes. **c** Distribution of retinoid polygenic risk score (PRS_Ret_), constructed using the most significantly associated *P-*value threshold, in the schizophrenia and healthy control cohorts. The black line represents the population mean for controls (left) and cases (right)

As five retinoid-related genes are located within haplotypes tagged by genome-wide significant schizophrenia associated SNPs, we hypothesised that other retinoid genes may also contribute to a wider polygenic signal without reaching genome-wide significance. Using MAGMA [30], *P*-values for SNPs from the PGC schizophrenia GWAS were aggregated in each member of our retinoid panel. Surprisingly, a number of these genes were nominally significant before correction (*P*_Raw_ < 0.05; Fig. 2a), implicating 17 genes in addition to the five uncovered in the SNP level GWAS (Supplementary Table 1). Notable genes included three retinoid receptors (*RARB*, *RARG* and *RXRB*), two aldehyde dehydrogenases (*ALDH1A2* and *ALDH1A3*), which catalyse the conversion of at-RA from its precursor, and the vitamin D receptor gene *VDR* – a retinoid X receptor binding partner. Analysis of public gene expression data for these 22 genes revealed they were upregulated relative to the rest of the retinoid panel in the brain, *P* = 0.0457 (Fig. 2b), with particular enrichment in the basal ganglia (*P* = 4.8 × 10^−3^) and amygdala (*P* = 0.0237, Supplementary Figure 1). Further, they demonstrated a greater degree of biological constraint as defined by predicted intolerance to loss-of-function variants (*P* = 2 × 10^−3^, two-sided Fisher’s exact test), and were overrepresented in de novo variant risk genes for neuropsychiatric disorders, as characterised in the NPdenovo database (*P* *=* 7.8 × 10^−3^, two-sided Fisher’s exact test, Supplementary Table 10).

### Retinoid PRS in associated with schizophrenia

We selected the 22 retinoid genes from the MAGMA analysis to test for association of PRS (PRS_Ret_) with schizophrenia and its severe cognitive subtype (CD), in which individuals have a greater degree of cognitive impairment [5]. PRS_Ret_ was enriched in schizophrenia cases at all *P*-value thresholds (*P*_T_) tested, with *P*_T_ = 1 × 10^−3^ the most significant adjusted for sex, population stratification and genome-wide PRS (*P* *=* 1.78 × 10^−4^, *χ*^2^ test of residual deviance). As residual deviance was significantly reduced in a model with both total PRS and PRS_Ret_ covariates compared with total PRS alone, it suggested that the retinoid signal is not a product of nonspecific inflation of total schizophrenia PRS in cases. At the same *P*_T_ as PRS_Ret_, total PRS depleted for the retinoid genes explained 6.79% of the variance on the liability scale, with PRS_Ret_ explaining 1.34%. However, when comparing the cognitive subtypes (CD vs CS), there was no enrichment of total schizophrenia PRS (lowest *P* = 0.715, *P*_T_ = 0.05) or PRS_Ret_ (lowest *P* = 0.224, *P*_T_ = 1 × 10^−3^) in CD cases. We then sought to identify participants with elevated PRS_Ret_, defined in this study as the top quartile of the ASRB cohort (Fig. 2c). As expected, high PRS_Ret_ was elevated in schizophrenia patients, with almost a third of that sample within this range (29.5%). In contrast, only 18.4% of non-psychiatric controls had an analogous PRS_Ret_.

### Rare variation in *RARB* and the CD subtype of schizophrenia

**Fig. 3:**
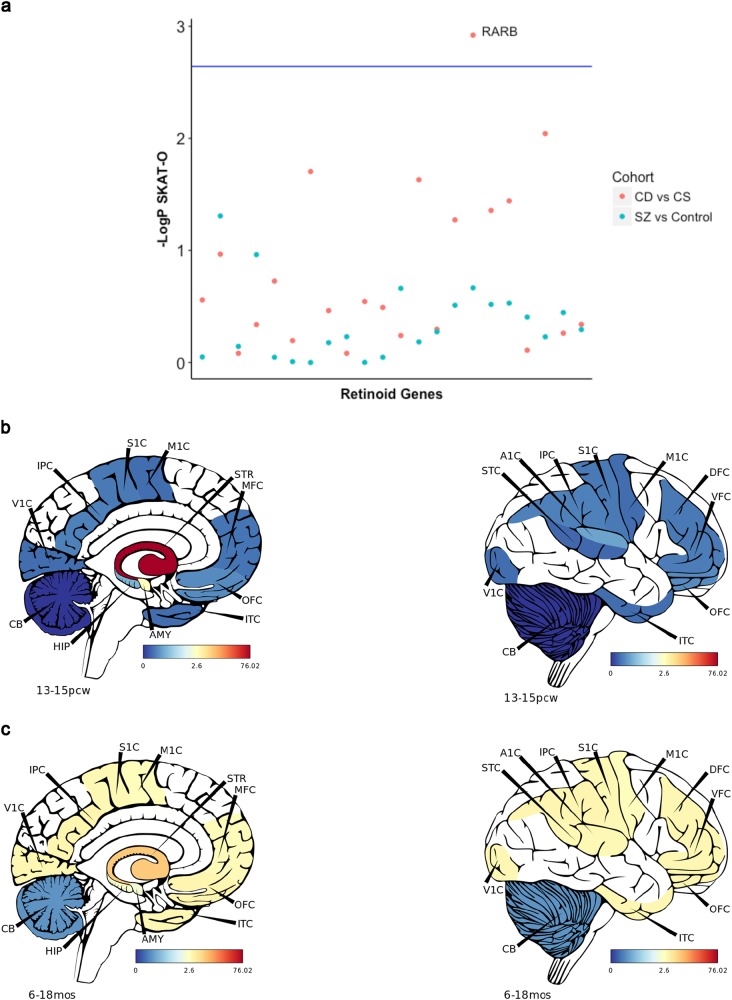
Rare variants in the retinoid receptor gene *RARB* enriched in a severe cognitive subtype of schizophrenia. **a** The combined effect of rare variants was tested at gene level using the SKAT-O test; analyses comparing cases with controls, and directly comparing cognitively spared [CS] with cognitive deficit [CD] cases of schizophrenia were conducted. Blue Bonferroni threshold corrected for the 22 genes tested for two phenotypes. **b, c** Expression of *RARB* in different brain regions from BrainSpan at (**b**) 13–15 weeks post conception (pcw) and (**c**) 6–18 months (mos) post-natal development. STC = caudal superior temporal cortex, A1C = primary auditory cortex, IPC = inferior parietal cortex, S1C = primary somatosensory cortex, M1C = primary motor cortex, DFC = dorsolateral prefrontal cortex, VFC = ventrolateral prefrontal cortex, OFC = orbital frontal cortex, ITC  =inferior temporal cortex, V1C = primary visual cortex, STR = striatum, MFC = medial prefrontal cortex, OFC = orbital frontal cortex, AMY = amygdala, HIP = hippocampus, CB = cerebellum

Common variants impacting the retinoid pathway were not specifically associated with CD status. We therefore speculated that rare variants mapped to the 22 retinoid genes tested in the PRS model might play a more significant role. We focused on rare loci (frequency < 0.01%) from participants who underwent WGS, the joint effect of such variants in each retinoid gene was then quantified in the entire schizophrenia cohort and in the CD subgroup only, respectively, using the SKAT-O framework [36]. No genes were associated with schizophrenia relative to healthy controls after correction for multiple testing (lowest: *ALDH1A3*, *P*_Raw_ = 0.035). However, the signal was much stronger in cases with CD, with a significant enrichment of rare variation in retinoic acid receptor beta gene *RARB* in the CD subtype compared with CS cases, *P*(FWE_Corr_) = 0.029 (Fig. 3a). Another retinoid receptor gene *RXRB*, the calcineurin subunit *PPP3CC* and the calreticulin gene *CALR* were nominally significant but exceeded the Bonferroni threshold (FWE_Corr_; Supplementary Table 2).

### *RARB* rare variant burden is associated with decreased cerebellar volume in CD individuals

**Fig. 4:**
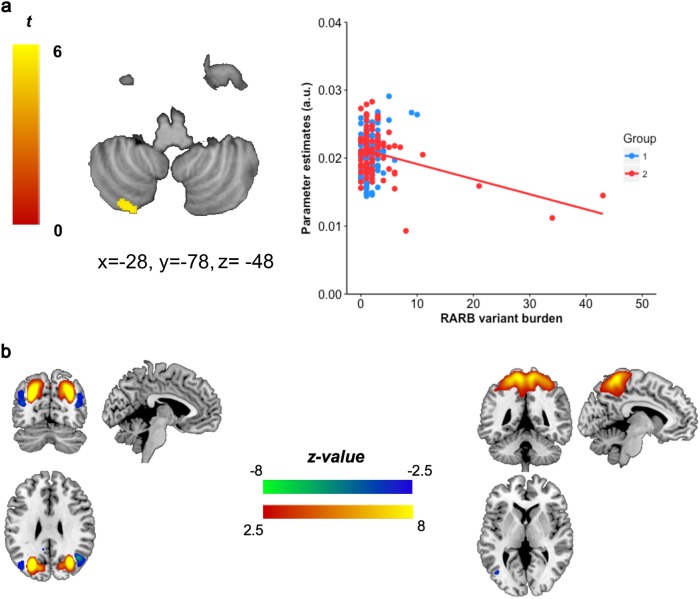
Association between *RARB* rare variant burden and neurophysiological phenotypes. **a** Interaction between *RARB* rare variant burden and volume (right) of a voxel cluster in the exterior of the left posterior cerebellum (left). Red points indicate the cognitive deficit (CD) subtype, whereas blue points represent cognitively spared (CS) schizophrenia cases. Coordinates of the significant cluster with reduced volume after voxel-wise correction (*x*, *y* and *z*) are stated. **b** Inter-subject covariance of grey matter significantly predicted by *RARB* rare variant burden in CD patients in regions outside the cerebellum. Source-based morphometry identified components (spatial regions) in which the covariation of grey matter concentration (GMC) was interrelated between individuals. *Z*-value indicates correlation between subjects, with a positive *Z*-value representing positively correlated inter-subject covariation and vice versa. Component C23: orange positive z-value region – precuneus, postcentral gyrus, paracentral lobule, sub-gyral, superior parietal lobule, inferior parietal lobule, medial frontal gyrus; blue negative z-value region – middle temporal ggyrus, inferior occipital gyrus. Component C8: orange positive z-value region – precuneus, sub-gyral, cuneus, superior parietal lobule; blue negative z-value region – middle temporal gyrus, angular gyrus, superior temporal gyrus

As *RARB* is enriched with rare variants only in the CD schizophrenia subtype, we investigated whether an increased burden of rare variation in this gene might be linked to specific neuroanatomical features. First, we established that there was no effect of cognitive subtype (CD/CS) on total GMV, WMV and CSF volume (MANCOVA; Wilk’s λ = 0.976, *F*_3,199_ = 1.611, *P* = 0.188, *η*_*p*_^2 ^= 0.024) among schizophrenia cases. No effect was also observed of cognitive group on GMV in any specific regions after voxel-wise correction. Notably, there was a significant interaction between *RARB* rare variant burden and the volume of a region in the left posterior cerebellum in CD cases after stringent voxel-wise correction – *P*(FWE_Corr_) = 0.023, MNI coordinates: *x* = −28, *y* = −78, *z* = −48, *k* = 95, *t*_200 _= 4.68, *z* = 4.55 (Fig. 4a).

We sought to investigate the effect of *RARB* rare variation on regions with correlated GMC using multivariate SBM [40]. *RARB* rare variant burden was significantly associated with covariation of GMC in the brain for CD cases (*R*^2^ = 0.638, *F*_43, 41_ = 1.680, *P* *=* 0.049). However, this signal was not significant in the CS cohort (*R*^2^ = 0.367, *F*_43, 41_ = 1.092, *P* *=* 0.360). Signals for this association (*P* < 0.05; Fig. 4b, Supplementary Table 12, Supplementary Table 14, Supplementary Figure 4) were found in components that encompass regions including the cerebellum, superior temporal gyrus and parietal lobe. Changes in shared covariation may therefore reflect hypervariability in grey matter between individuals in these regions, predicted by rare variants in *RARB* in CD cases.

### Retinoid receptor binding sites (DR5-RARE) in schizophrenia

The functional impact of at-RA is largely conferred by its capability to modulate the expression of numerous target genes throughout the genome. Retinoid receptors dimerise and bind to DNA motifs termed RAREs, most commonly DR5-RARE. We analysed if variation that alter DR5-RARE motifs, and thus may modify the binding dynamics of retinoid receptors, plays a role in schizophrenia. Common SNPs from the PGC schizophrenia GWAS, which overlap a DR5-RARE were extracted, however, none were genome-wide significant. Using the WGS cohort, rare variants were extracted from 3252 autosomal DR5-RARE coordinates. In total, 156 rare variants were identified (*N*_Cases_ = 118, *N*_Controls_ = 38), which were overrepresented in affected individuals (*P* = 0.0226, two-sided Fisher’s exact test).

In addition, we investigated if genes within 10 kb of an in silico predicted DR5-RARE (*N*_Unique Genes_ = 2998) were differentially expressed in publicly available summary statistics of two RNA sequencing (RNAseq) studies in schizophrenia [12]. Cases and control samples of post-mortem dorsolateral prefrontal cortex (DLPFC) sequenced by the CommonMind consortium, as described elsewhere [17], displayed 76 differentially expressed genes proximal to predicted DR5-RAREs (Supplementary Figure 2). These genes were enriched for eight pathways after correction for multiple testing (*q* < 0.05), several of which are involved in neurodevelopment including *axon guidance* (*P* = 5.63 × 10^−4^, *q* = 0.0257) and *neural cell adhesion molecule* *signalling during neurite outgrowth* (*P* = 2.06 × 10^−3^, *q* = 0.0449, Supplementary Table 3). This approach was also applied to the differentially expressed genes in a large case–control study of schizophrenia in lymphoblastoid cell lines (LCLs), with 151 DR5-RARE proximal genes dysregulated [41]. In this instance, 27 pathways were enriched for predominantly immune-related functions such as *Measles* (*P* *=* 2.391 × 10^−5^, *q* = 8.01 × 10^−3^) and *MHC (Major Histocompatibility Complex) class II antigen presentation* (*P* = 1.87 × 10^−3^, *q* = 0.0369, Supplementary Table 4).

## Discussion

Deficits in neuronal connectivity are widely believed to be involved in the neuropathology of schizophrenia [42]. As functional relationships between neurons are immensely complex, disturbances in this architecture are likely to have highly variable consequences for individuals and may explain some of the phenotypic heterogeneity of the disorder. In particular, severe CDs have been linked to neuroanatomical phenotypes, including cerebral grey matter loss [7, 43]. The aetiology of dysconnectivity in schizophrenia is thought to be conferred by a combination of heritable factors and environmental insults [44–46]. Retinoid signalling, in particular, is critical for neuronal differentiation and provides a nexus between neurodevelopment in utero and the regulation of intrinsic pathways to psychiatric illness, including synaptic plasticity, dopaminergic signalling and inflammation [47, 48]. This has been supported by recent studies of the common variant genetics of schizophrenia, with five genome-wide significant loci potentially implicating retinoid-related genes. In our study, we expanded on this initial finding to elucidate a larger polygenic signal spanning 22 genes in the retinoid pathway. We then profiled polygenic risk within this geneset (PRS_Ret_) in a case–control cohort and demonstrated enrichment in schizophrenia.

This enrichment of retinoid-related common variant burden in the disorder not only supports the neurodevelopmental hypothesis but could be pharmacologically modulated, with evidence of efficacy for exogeneous retinoids as a schizophrenia treatment adjuvant [19, 20]. Dietary modulation via retinol fortified foods or vegetables high in the retinol precursor beta-carotene may also be beneficial, particularly as intake of beta-carotene has been shown to be low in some schizophrenia cohorts [49]. The preliminary data from the ASRB cohort indicated that a clinically significant proportion of patients may be affected by this common variant burden, which is elevated PRS_Ret_, warranting testing of potential therapeutic interventions relating to this system.

As CD reduces fecundity and is subject to natural selection [50, 51], we suspected that rare variation may be enriched in CD schizophrenia, as it is in individuals with autism spectrum disorder and intellectual disability [33]. This is consistent with the observation of excess low-frequency coding variation in cases of schizophrenia with comorbid intellectual disability [52]. We expand this here by demonstrating the rare variation in *RARB* is associated with marked cognitive impairment (CD subtype) in schizophrenia. The biological implications of *RARB* for cognition is evidenced by both human studies and animal models. *RARB* is biologically constrained by natural selection; extreme phenotypes attributed to rare exonic variants in this gene include intellectual disability, spasticity and congenital ocular defects [53]. Further, *RARB* is implicated in several neurodevelopmental processes, including formation of the blood–brain barrier and striatonigral GABAergic populations [54, 55]. Murine *RARB* knock-out models reveal its intrinsic link to cognitive function, as both long-term potentiation and depression, which underlie hippocampal plasticity, are functionally ablated [56]. BrainSpan data, an atlas of the developmental transcriptome, indicates that cortical expression of *RARB* peaks in the post-natal period (Fig. 3c), whereas this occurs in utero within the striatum (Fig. 3b) [37]. This spatiotemporal transcription profile suggests that compromised *RARB* function may contribute to aberrant dopaminergic signalling, which occurs in schizophrenia, but may also impair normal cognitive development in early life. *RARB* involvement in schizophrenia more broadly is supported by evidence of increased burden of common variation in this gene. This suggests subtle alteration of *RARB* function, independent of cognitive status, is involved in the pathophysiology of schizophrenia but with likely less magnitude than rare variation.

Distinct neuroanatomical features of schizophrenia have often been difficult to link to genomic risk. This problem is compounded in rare variant analyses of the non-coding genome, as loci have highly heterogeneous impacts depending on the local epigenome and other distal regulatory factors. However, in spite of these challenges we observed evidence of structural brain phenotypes associated with the burden of rare *RARB* variation in the CD subtype. Decreased cerebellar volume in its left posterior region was associated with greater *RARB* rare variant burden in CD schizophrenia after stringent voxel-wise correction. Mounting evidence implicates the cerebellum in cognitive processes, particularly as there are multiple functional connections with cortical regions [57, 58]. Lesion studies suggest that the cerebellar posterior lobe plays a role in cognition as opposed to the predominant role of the anterior lobe in motor functions [59]. Specifically, the right posterior lobe is associated with language, whereas its left posterior regions are linked with visuospatial processing [60, 61]. Reduced cerebellar volume, along with several other abnormalities, is associated with schizophrenia as reviewed by Andreasen and Pierson [62]. Given the diverse spectrum of genes and pathways modulated by *RARB*, it is possible that the cerebellum is more physiologically susceptible to comprehensive neuronal retinoid dysregulation, appearing as a ‘sentinel node’ for the anatomical effects of *RARB* disruption. This is supported by our SBM analyses where increasing *RARB* rare variant burden could predict the covariation of GMC in the brain among CD cases. Moreover, animal models of neuronal development also implicate vulnerability of the cerebellum and other dorsally derived hindbrain neurons to retinoid dysregulation [63]. Further functional dissection of intergenic and other non-coding regions in and around *RARB* will help with deciphering the mode of action of this rare variant burden.

To better understand the biological implications of altered retinoid signalling in schizophrenia, the role of at-RA regulated target genes needs to be refined. Retinoid disruption may impact expression of proximal genes to RAREs, with implications for a diverse range of systems. We found DR5-RARE proximal genes whose expression was altered in schizophrenia were enriched within relevant pathways to psychiatric disorders in RNAseq data from DLPFC and LCL samples. Although pathways involved in neurodevelopment, such as axon guidance, have clear significance for schizophrenia, others – particularly systemic inflammation – have also been reported in the disorder, especially among cases with CDs [64, 65]. This provides notable evidence of downstream retinoid dysregulation in two independent schizophrenia cohorts. Variants within non-coding DR5-RARE sequences present as an alternate mechanism to alter the signalling capability of at-RA. The preliminary evidence for rare DR5-RARE variant enrichment in schizophrenia from this study suggests that highly complex biological consequences may arise from disturbances in the retinoid pathway. Rare variants that alter receptor binding dynamic will likely have a different impact depending on the target transcript function and the extent of DR5-RARE disruption. Investigation into the response of retinoid receptor binding to motif variation will be needed to determine the precise neurobiological implications of these variants.

The integration of genomics with cognitive and neuroanatomical phenotypes facilitated a more detailed examination of putative impacts of variation in retinoid loci in this study, however, there are some important limitations to acknowledge. First, although we aimed to thoroughly investigate retinoid biology, the system is immensely complex and there are many emerging aspects, which will need be addressed in future analyses. This includes several retinoid associated genes and a suite of other putative target genes with poorly characterised RARE motifs and structural configurations with  variable nucleotide repeat lengths [12, 66]. These in silico motif predictions will also ideally be accompanied by more extensive in vitro validation in ne urologically relevant cell types. Further, although the rare variant association of *RARB* is strong, particularly in the context of this sample size, this requires independent replication of both the genomic effect in cases with severe CDs, along with the observed neuroanatomical phenotype.

In summary, we demonstrated a common variant polygenic signal impacting retinoid genes in schizophrenia, with evidence of rare variation disproportionately affecting cases with severe CD. With signs of efficacy in retinoid-based treatments of schizophrenia, the findings reported have significant clinical implications and may be clinically actionable. Prior trials of the retinoid X receptor agonist Bexarotene have proven efficacious despite testing in genotypic undifferentiated cohorts [19, 20]. We propose that determination of genetic liability to retinoid dysfunction in individuals could be used to target this and other retinoid-based interventions with more precision and, thus, achieve a higher therapeutic yield. The value of this approach is supported by the large proportion of our schizophrenia cohort with elevated PRS_Ret_. Alternatively, as rare variation may impact at-RA signalling in a subset of CD individuals with greater magnitude, future trials of retinoid compounds and analogues should also assess the responsiveness of cognitive symptoms.

## Electronic supplementary material

Supplementary Tables 1-4

Supplementary Tables 12-15

Supplementary Material
