# Supplementary material for: Polygenic disruption of retinoid signalling in schizophrenia and a severe cognitive deficit subtype
Source: Mol Psychiatry. 2018 Dec 7;25(4):719–31. doi: 10.1038/s41380-018-0305-0 (PMC7156344; doi:10.1038/s41380-018-0305-0)
Supplement: Supplementary file 3 — Supplementary Material [file 41380_2018_305_MOESM3_ESM.pdf]

SUPPLEMENTARY MATERIALS

Polygenic disruption of retinoid signalling in schizophrenia and its severe cognitive deficit symptoms

CONTENTS

Supplementary Note 1	2
Supplementary Note 2	2
Supplementary Note 3	2
Supplementary Note 4	3
Supplementary Note 5	5
Supplementary Table 5	6
Supplementary Table 6	6
Supplementary Table 7	7
Supplementary Table 8	7
Supplementary Table 9	8
Supplementary Table 10	8
Supplementary Table 11	9
Supplementary Figure 1	10
Supplementary Figure 2	11
Supplementary Figure 3	12
Supplementary Figure 4	13
Supplementary Figure 5	15
References	16

Supplementary Note 1

The quality control procedure for samples genotyped by SNP array in the ASRB cohort are as follows. Before imputation genotyped autosomal SNPs were removed if they failed a series of quality control (QC) criteria implemented with PLINK 1.9: MAF < 0.01, call rate < 98% and Hardy-Weinberg Equilibrium (HWE) $P < 10^{-6}$. Ambiguous SNPs not assigned to a strand were also filtered out along with all non-autosomal sites. Individuals with discordant sex information were excluded from the cohort along with one individual of a pair with an Inbreeding Coefficient (IF) > 0.2. High quality autosomal SNPs (MAF > 0.05) in relative linkage equilibrium ($R^2 < 0.1$) were input for relatedness testing, whilst regions of long range linkage disequilibrium were removed (1). Individuals with high relatedness (N=9) defined by genome wide identity by state (pi_hat > 0.185) were removed. Principal components analysis (PCA) was also undertaken with PLINK 1.9. Population outliers were excluded (N=63) using k-means clustering, wherein the five generated clusters represented the super-populations in the 1000 Genomes Phase 3 reference panel (Supplementary Figure 3)

Supplementary Note 2

The cohort described previously was sequenced at the Garvan Institute of Medical Research: Kinghorn Centre for Clinical Genomics using the Illumina HiSeq X Ten system. After library and cluster generation using standard Illumina protocols, 1μg of high integrity peripheral blood mononucleocyte DNA was sequenced with 2 x 150 base pair paired-end reads, such that 75% of bases had a Phred quality score greater than 30 and a minimum mean yield of 30x coverage.

Supplementary Note 3

To investigate the impact of genomic variation on at-RA signalling in schizophrenia we constructed a panel of 107 retinoid genes based on gene ontology and the wider literature (Supplementary Table 1). Retinoid related gene sets were sourced from the molecular signatures database (MSigDB) by searching the following keywords: 'retinoic acid', 'retinoid', 'retinol' and 'vitamin A' (2). This gene list was supplemented with the five genes encoding proteins comprising the calcineurin complex, which have recently been demonstrated to play an important role in at-RA mediated synaptic plasticity (3).

Supplementary Note 4

Image pre-processing

High-resolution T1-weighted structural MRI scans (MPRAGE; 176 contiguous 1mm sagittal slices; field-of-view 250 x 250 mm2, time-to-repetition 1980ms, time-to-echo 4.3ms, data acquisition matrix 256 x 256, voxel size 0.98 x 0.98 x 1.0 mm3, flip angle 15°) were collected on Siemens Avanto 1.5T scanners across five Australian research sites by the ASRB (4). In the WGS cohort, 210 cases of schizophrenia with assigned cognitive subphenotype had MRI scans available that passed a stringent quality control (Supplementary Table 11). The distribution of usable scans was statistically uniform across scanning site locations when comparing CS and CD cases ($\chi^2 = 3.959$, df = 4, $P = 0.412$). Quality assessment procedures included radiologist inspection for gross and/or pathological brain abnormalities, followed by an automated quality assessment as described previously (5). Total intracranial volume (TIV), total grey matter (GM), total white matter (WM) and total cerebrospinal fluid (CSF) volumes were then extracted using the Computational Anatomy Toolbox (CAT12, v1073; Structural Brain Mapping group, Jena University Hospital, Jena, Germany; http://dbm.neuro.uni-jena.de/cat/index.html) for SPM12 (v6906; Wellcome Trust Centre for Neuroimaging, London, UK; http://www.fil.ion.ucl.ac.uk/spm) in MATLAB r2013a (Mathworks Inc., Sherborn, MA, USA).

Voxel-based morphometry (VBM)

The voxel-based morphometry (VBM) pipeline normalised scans to a standard Montreal Neurological Institute (MNI) template using the SPM's 'Diffeomorphic Anatomic Registration through Exponentiated lie' (DARTEL) algebra normalisation (6), this was followed by segmentation into GM, WM and CSF. In addition, partial volume effects (7), hidden Markov Random Field model (8) and adaptive maximum *a posteriori* estimations (9) were applied to the segmentation. Following these steps, an additional quality control on sample homogeneity was performed to ensure there were no outlier scans with a Mahalanobis distance between mean correlations and weighted overall image quality significantly higher than the other scans. Finally, images were smoothed with an 8mm full width at half maximum (FWHM) Gaussian kernel. Individual pre-processed images (smoothed modulated images) were used in second-level (groups) VBM and SBM analyses. The effect of cognitive

group (CD/CS) was on total GM, WM and CSF measures was investigated using multivariate analysis of covariance (MANCOVA). All imaging analyses were adjusted for age, sex, total intracranial volume (TIV) and scanning location for the MRI. Individual count of *RARB* rare variants was conducted using the allelic scoring flag (--score) in PLINK 1.9. A hierarchical multiple regression was conducted on whole brain-volumes with cognitive group, *RARB* rare variant burden and their interaction as independent variables. Global scaling was used with the TIV option to reduce the effect of orthogonality due to strong correlation between TIV and the other model covariates, implicating a change in the absolute threshold for masking (0.2/30 = 0.007). Statistical inference was made using a stringent voxel-level threshold of $P < 0.05$ (family-wise error (FWE)-corrected $P(\text{FWE}_{\text{Corr}}) < 0.05$). Only clusters larger than 30 voxels (around 1cm^3) were considered and the significant region identified mapped with CAT12 using the neuromorphometrics atlas in DARTEL space.

Source-based morphometry (SBM)

The protocol for SBM is outlined in detail in Xu *et al.* (10). Independent components analysis (ICA) was undertaken on grey matter images after the pre-processing steps described above for a large ASRB cohort comprising 337 schizophrenia cases and 193 healthy controls from which our imaging subset is derived. Briefly, independent components analysis (ICA) was conducted on pre-processed grey matter images, using the Infomax algorithm as part of the "Group ICA for fMRI Toolbox" (GIFT v4.0a; http://mialab.mrn.org/software/gift). The number of independent components was estimated using the 'minimum description length' criteria on the ASRB cohort (11). Each grey matter image was converted to a one-dimensional vector and arrayed into one 530-row, subjects-by-voxels matrix. The subjects-by-voxels matrix was composed of *i)* a mixing matrix representing the contribution of each subject for a given component (loading coefficients), and *ii)* a source matrix representing the relationship between components and brain voxels. The ICASSO algorithm was used (20 runs) with bootstrapping and permutation to increase the stability of the estimated components (12). The minimum description length criteria estimated 36 independent components (C1-36; Supplementary Table 13). Following ICA, loading coefficients from the mixing matrix were extracted for investigation for the subsample of interest. To visualise the components, the source matrix was reshaped to a three-dimensional image, scaled to unit of standard deviations (z-maps) and the threshold was set to $|z|>2.5$, with a minimum cluster size of 1cm^3. Maps for each significant component were overlaid on an MNI template, and

stereotaxic coordinates from clusters above a $z>2.5$ threshold were obtained using the Talairach Daemon database (http://www.talairach.org/daemon.html).

We sought to identify the ICA components which were associated with *RARB* rare variant burden in each cognitive subtype. A series of hierarchical regressions was performed with *RARB* variant burden as the dependent variable, the covariates we adjusted for in the VBM model were then entered in the first step, followed in the second step by the 36 ICA components as independent variables. The interpretation of the impact of *RARB* variant burden on GMC covariation for each component depends both on the voxels which comprise the spatial component and the sign of the loading coefficient. If the regression coefficient (standardised β) is positive, the greater the *RARB* rare variant burden, the more the loading coefficient is positive, and if the spatial component is predominantly made of 'positively correlated voxels', then *RARB* burden predicts increased grey matter concentration (GMC) covariation in these regions for that particular spatial component. If the spatial component is predominantly made of 'negatively correlated voxels', then *RARB* burden predicts decreased GMC covariation in these regions. Conversely, if the regression coefficient is negative (greater *RARB* rare variant burden results in a more negative loading coefficient), and if the spatial component is predominantly made of 'positively correlated voxels', then *RARB* burden is considered predicting deceased GMC covariation in these regions for that particular spatial component; if the spatial component is predominantly made of 'negatively correlated voxels', then *RARB* burden increased GMC covariation in these regions. As previously demonstrated, a higher loading coefficient indicates that the spatial pattern is more strongly weighted in the data (13).

Supplementary Note 5

Genes within 10 kb of a predicted DR5-RARE were sourced from the summary statistics of two published RNAseq studies in schizophrenia. Firstly, the publicly available list of differentially expressed genes (DEGs) in the DL-PFC from Fromer *et al.* (14) was obtained from the CommonMind consortium (Synapse ID: syn5607603). The list of DEGs in a large LCL study were downloaded from the supplementary materials of that publication (15). Pathways analysis on DR5-RARE proximal DEGs was undertaken using ConsensusPathDB utilising pathway-based sets (16). Significant pathways were defined as those in either tissue with a q-value < 0.05.

Supplementary Tables 5 – 11

Supplementary Table 5. Cohort size and sex composition of the entire case and control cohort genotyped via SNP array from the Australian Schizophrenia Research Bank (ASRB).

	Control	Case
Total	251	425
Males	110	283
Females	141	142
Mean Age (s.d.)[1]	39.5 (13.40)	39.88 (10.92)

[1]s.d. = standard deviation

Supplementary Table 6. Cohort size and sex composition of the entire case cohort who were subjected to cognitive suphenotyping, and controls genotyped via SNP array from the Australian Schizophrenia Research Bank (ASRB).

	Control	Case	
		CD	CS
Total	251	171	221
Males	110	126	134
Females	141	45	87
Mean Age (s.d.)[1]	39.50 (13.40)	38.75 (10.44)	40.81 (11.34)
Mean GAF[2] (s.d.)	84.13 (8.81)	48.57 (11.92)	57.39 (12.75)
Mean Onset Age (s.d.)	N/A	23.25 (6.91)	24.14 (7.06)

[1]s.d. = standard deviation
[2]GAF = Global Assessment of Functioning scale, a lower score indicates greater symptom severity for the disorder

Supplementary Table 7. Cohort size and sex composition of the entire case and control cohort who underwent whole genome sequencing from the Australian Schizophrenia Research Bank (ASRB).

	Control	Case	
		CD	CS
Total	148	163	158
Males	72	103	111
Females	76	47	55
Mean Age (s.d.)[1]	40.73 (13.36)	38.95 (10.14)	39.98 (11.22)
Mean GAF[2] (s.d.)	84.89 (9.57)	49.09 (12.76)	57.55 (12.53)
Mean Onset Age (s.d.)	N/A	23.22 (6.82)	23.51 (6.12)

[1]s.d. = standard deviation
[2]GAF = Global Assessment of Functioning scale, a lower score indicates greater symptom severity for the disorder

Supplementary Table 8. Sample characteristics of the MRI imaging cohort for *RARB* variant burden analyses

	Age	Sex(M/F)	GMV[1]	WMV[2]	CSF[3]	TIV[4]
CS (N=125)	39.06 (11.15)	88/37	705.13 (77.46)	551.29 (62.10)	348.52 (64.61)	1604.94 (148.97)
CS (N=85)	36.99 (9.09)	63/22	692.56 (65.79)	544.16 (67.04)	344.88 (63.38)	1581.61 (143.14)
Significance	*P*=0.140	*P*=0.640	*P*=0.202	*P*=0.430	*P*=0.687	*P*=0.259

Mean and standard deviation reported
[1]Grey matter volume (GMV)
[2]White matter volume (WMV)
[3]Cerebrospinal fluid volume (CSF)
[4]Total intracranial volume (TIV)

Supplementary Table 9. Cognitive measures utilised to derive to cognitive subtypes in schizophrenia used for this study.

Cognitive Domain	Abbreviation
Wechsler Test of Adult Reading	WTAR
Wechsler Abbreviated Scale for Intelligence	WASI
Controlled Oral Word Association Test	COWAT
Letter Number Sequencing (Scaled Score)	LNS
Repeatable Battery for the Assessment of Neuropsychological Status – immediate memory	RBANS – immediate memory
Repeatable Battery for the Assessment of Neuropsychological Status – constructional	RBANS – constructional
Repeatable Battery for the Assessment of Neuropsychological Status – language	RBANS – language
Repeatable Battery for the Assessment of Neuropsychological Status – attention	RBANS – attention
Repeatable Battery for the Assessment of Neuropsychological Status – delayed memory	RBANS – delayed memory
Repeatable Battery for the Assessment of Neuropsychological Status – total scaled score	RBANS – total scaled score

[1]Study parameters described in detail in Green *et al*. (17)

Supplementary Table 10. Retinoid genes implicated as *de novo* risk genes for neuropsychiatric disorders in the NPdenovo database

Gene ID	Association Level[1]	P value	Neuropsychiatric Disorder
ZNF536	Possible	0.0235	Schizophrenia
RAI1	Positive	1.243×10^{-3}	Intellectual Disability
NR4A2	Possible	0.0124	Autism Spectrum Disorder
NR1H2	Positive	3.201×10^{-3}	Epileptic Encephalopathy
PML	Possible	0.01931	Schizophrenia
RARG	Positive	1.396×10^{-3}	Schizophrenia
RBP3	Possible	0.02342	Schizophrenia

[1]Association level as defined by the NPdenovo (neuropsychiatric *de novo* mutation database)

Supplementary Table 11. Quality filtering thresholds for sequenced rare SNVs

Parameter	Abbreviation	Filtering Threshold
QualByDepth	QD	< 2.0
FisherStrand	FS	> 60.0
RMSMappingQuality	MQ	< 40.0
MappingQualityRankSumTest	MQRankSum	< -12.5
RedPosRankSumTest	RedPosRankSum	< -8.0
StrandOddsRatio	SOR	> 3.0

Supplementary Figures

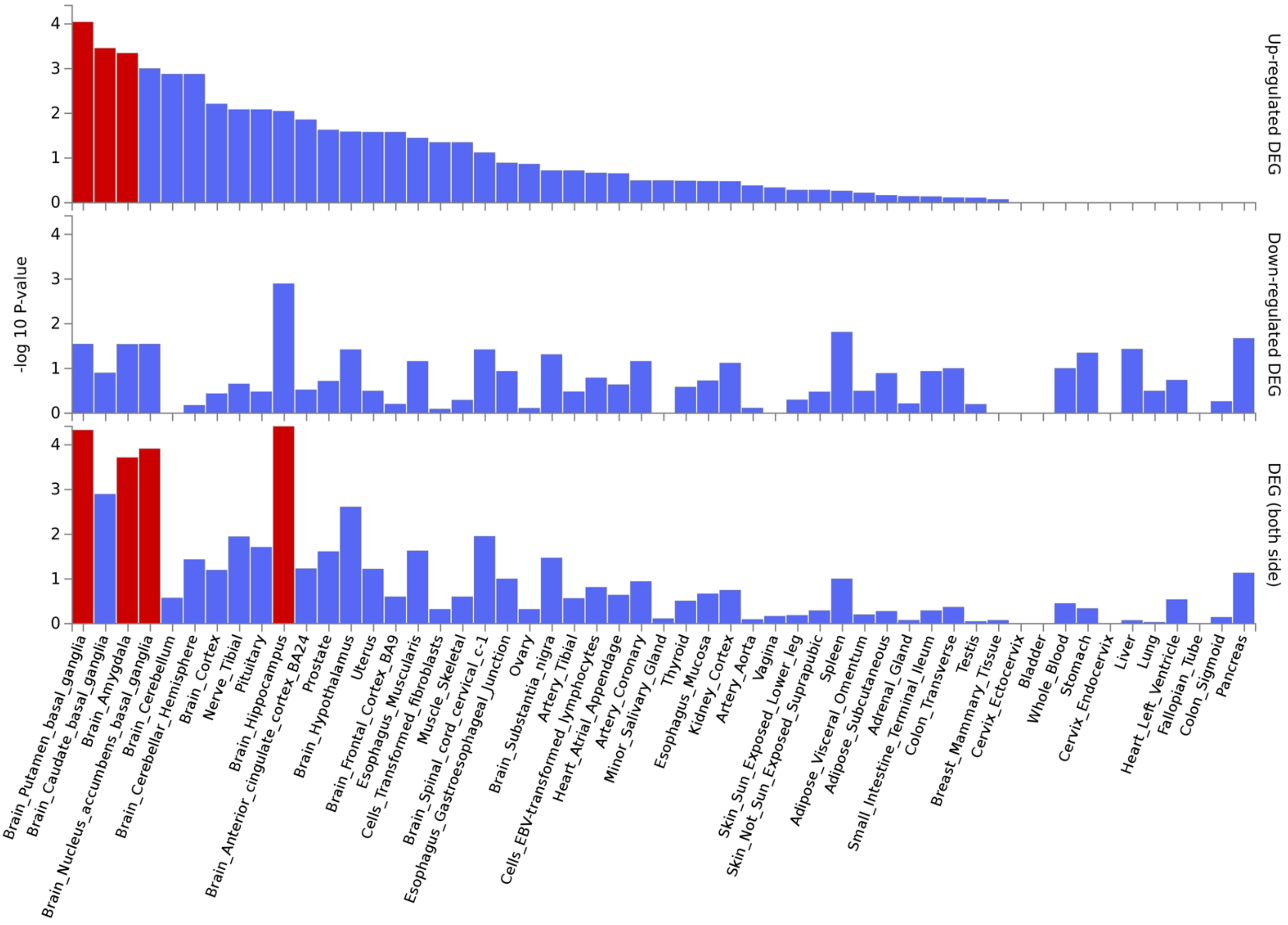

Supplementary Figure 1. Tissue specific expression in GTEx 50 tissue types of retinoid polygenic risk genes relative to the rest of the panel. Red bars indicate that the 22 genes (MAGMA $P < 0.05$) are significantly differentially expressed after multiple testing correction in that tissue relative to the rest of the retinoid panel as background. A one-sided test for both upregulation and downregulation is reported along with a two-sided test. DEG = Differentially Expressed Gene.

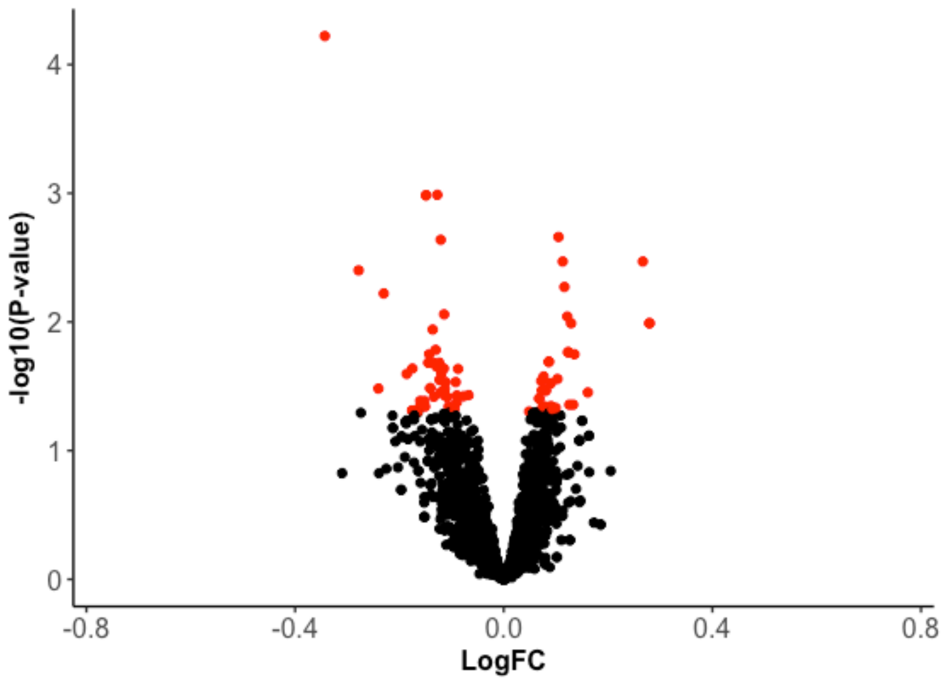

Supplementary Figure 2. Volcano plot of genes proximal to a predicted DR5-RARE in the CommonMind DLPFC RNAseq study. Genes are within 10 kilobases of an *in silico* predicted DR5-RARE retinoid receptor binding site, for which differential expression analysis in schizophrenia was undertaken in the dorsolateral pre-frontal cortex (DLPFC) by the CommonMind Consortium. Points highlighted red the 76 are significant differentially expressed genes after the application of multiple testing correction [as described in Frommer *et al.* (12)].

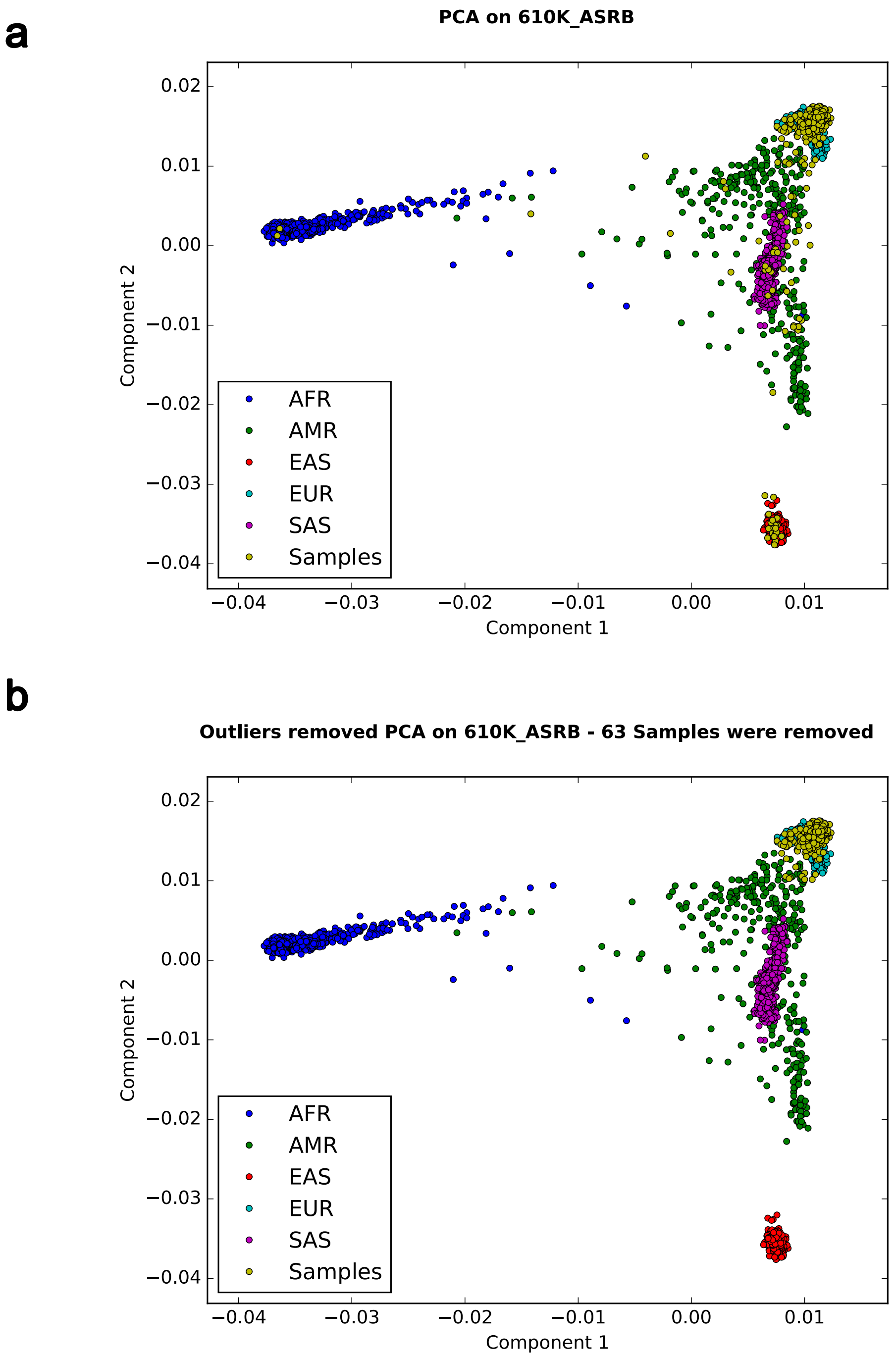

Supplementary Figure 3. Removal of population outliers from the ASRB 610K array genotyped cohort using principal components analysis. Clustering by each superpopulation using the 1000 genomes phase 3 reference panel, 63 population outliers were removed. AFR = African, AMR = American, EAS = East Asian, EUR = European, SAS = South East Asian, Samples = ASRB 610K array cohort. (**a**) Clustering before removal of population outliers. (**b**) Population outliers removed.

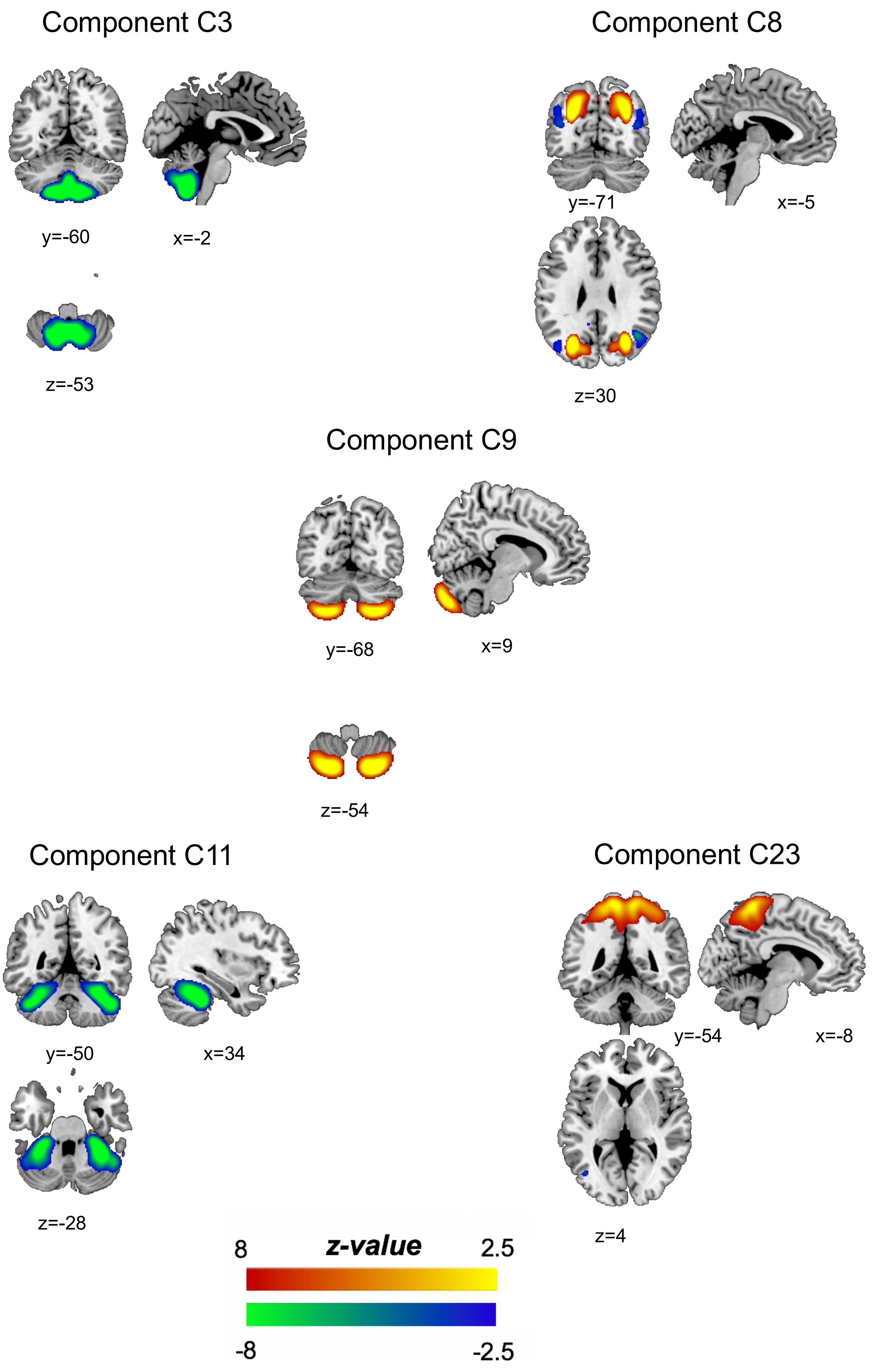
Component C3
y=-60
x=-2
z=-53
Component C8
y=-71
x=-5
z=30
Component C9
y=-68
x=9
z=-54
Component C11
y=-50
x=34
z=-28
Component C23
y=-54
x=-8
z=4
z-value
8
2.5
-8
-2.5

Supplementary Figure 4. Independent spatial components whose grey matter concentration (GMC) covariance is associated with increasing *RARB* rare variant burden in cognitive deficit (CD) schizophrenia subtype cases. The anatomical definitions of each of the five significantly associated components from the source-based morphometry (SBM) are outlined in detail in supplementary table 12. Effect of *RARB* rare variant burden on GMC covariance depends on the sign of the loading coefficient (whether the regression coefficient is positive or negative) and the composition of the spatial component. Components with positive z-scores are comprised of positively correlated voxels in terms of the inter-subject relationship of GMC (yellow/orange), whilst negative z-scores are comprised of negatively correlated voxels in terms GMC (blue/green).

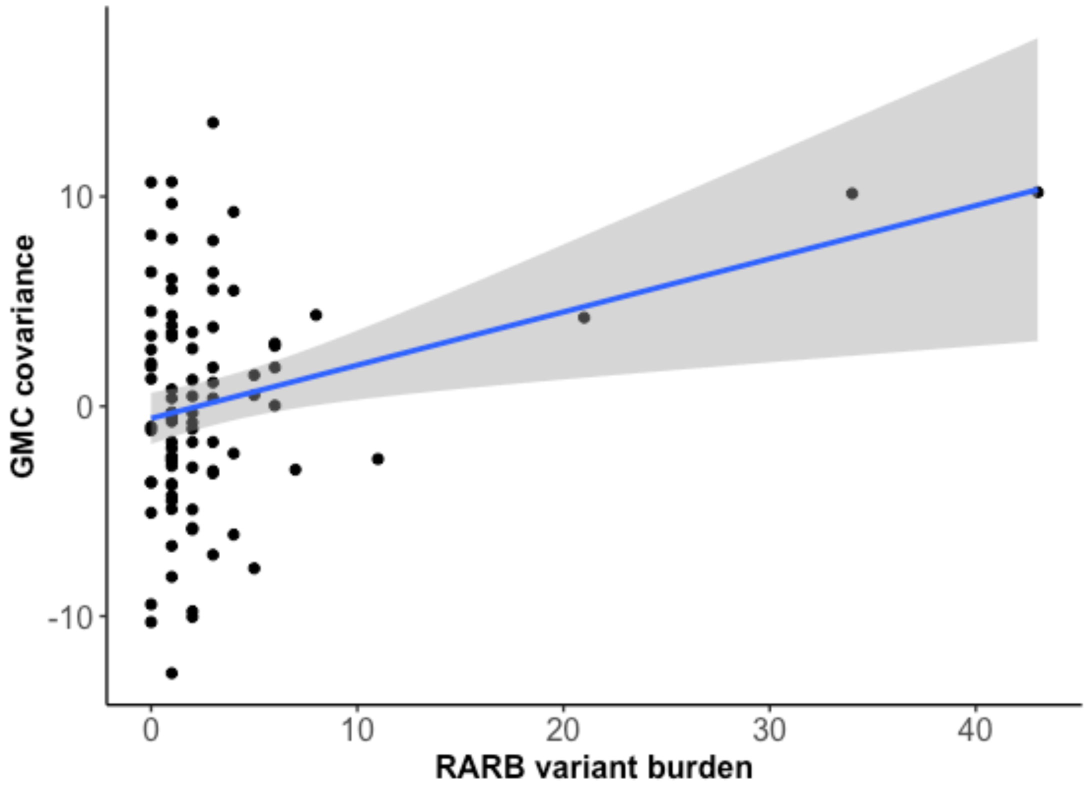

Supplementary Figure 5. Effect of increasing burden of rare *RARB* variation of covariation of grey matter concentration (GMC) throughout the brain in CD subtype individuals. Relationship between burden of rare variants in the retinoic acid receptor beta gene *RARB* and the combined covariation of grey matter concentration within 36 interrelated brain regions (derived by independent components analysis [ICA]).

References

1. Price AL, Weale ME, Patterson N, Myers SR, Need AC, Shianna KV, et al. Long-Range LD Can Confound Genome Scans in Admixed Populations. American Journal of Human Genetics. 2008;83(1):132-5.
2. Liberzon A, Birger C, Thorvaldsdóttir H, Ghandi M, Mesirov Jill P, Tamayo P. The Molecular Signatures Database Hallmark Gene Set Collection. Cell Systems.1(6):417-25.
3. Arendt KL, Zhang Z, Ganesan S, Hintze M, Shin MM, Tang Y, et al. Calcineurin mediates homeostatic synaptic plasticity by regulating retinoic acid synthesis. Proceedings of the National Academy of Sciences. 2015;112(42):E5744-E52.
4. Loughland C, Draganic D, McCabe K, Richards J, Nasir A, Allen J, et al. Australian Schizophrenia Research Bank: a database of comprehensive clinical, endophenotypic and genetic data for aetiological studies of schizophrenia. The Australian and New Zealand journal of psychiatry. 2010;44(11):1029-35.
5. Quidé Y, Matosin N, Atkins JR, Fitzsimmons C, Cairns MJ, Carr VJ, et al. Common variation in ZNF804A (rs1344706) is not associated with brain morphometry in schizophrenia or healthy participants. Progress in Neuro-Psychopharmacology and Biological Psychiatry. 2018;82:12-20.
6. Ashburner J. A fast diffeomorphic image registration algorithm. NeuroImage. 2007;38(1):95-113.
7. Tohka J, Zijdenbos A, Evans A. Fast and robust parameter estimation for statistical partial volume models in brain MRI. NeuroImage. 2004;23(1):84-97.
8. Cuadra MB, Cammoun L, Butz T, Cuisenaire O, Thiran JP. Comparison and validation of tissue modelization and statistical classification methods in T1-weighted MR brain images. IEEE transactions on medical imaging. 2005;24(12):1548-65.
9. Rajapakse JC, Giedd JN, Rapoport JL. Statistical approach to segmentation of single-channel cerebral MR images. IEEE transactions on medical imaging. 1997;16(2):176-86.
10. Xu L, Groth KM, Pearlson G, Schretlen DJ, Calhoun VD. Source-Based Morphometry: The Use of Independent Component Analysis to Identify Gray Matter Differences With Application to Schizophrenia. Human brain mapping. 2009;30(3):711-24.
11. Li Y-O, Adalı T, Calhoun VD. Estimating the number of independent components for functional magnetic resonance imaging data. Human Brain Mapping. 2007;28(11):1251-66.
12. Himberg J, Hyvarinen A, Esposito F. Validating the independent components of neuroimaging time series via clustering and visualization. Neuroimage. 2004;22(3):1214-22.
13. Gupta CN, Calhoun VD, Rachakonda S, Chen J, Patel V, Liu J, et al. Patterns of Gray Matter Abnormalities in Schizophrenia Based on an International Mega-analysis. Schizophr Bull. 2015;41(5):1133-42.
14. Fromer M, Roussos P, Sieberts SK, Johnson JS, Kavanagh DH, Perumal TM, et al. Gene Expression Elucidates Functional Impact of Polygenic Risk for Schizophrenia. Nature neuroscience. 2016;19(11):1442-53.
15. Sanders AR, Drigalenko EI, Duan J, Moy W, Freda J, Göring HHH, et al. Transcriptome sequencing study implicates immune-related genes differentially expressed in schizophrenia: new data and a meta-analysis. Translational Psychiatry. 2017;7(4):e1093.
16. Herwig R, Hardt C, Lienhard M, Kamburov A. Analyzing and interpreting genome data at the network level with ConsensusPathDB. Nature Protocols. 2016;11:1889.
17. Green MJ, Cairns MJ, Wu J, Dragovic M, Jablensky A, Tooney PA, et al. Genome-wide supported variant MIR137 and severe negative symptoms predict membership of an impaired cognitive subtype of schizophrenia. Molecular Psychiatry. 2012;18:774.
